# Research on sustainable green building space design model integrating IoT technology

**DOI:** 10.1371/journal.pone.0298982

**Published:** 2024-04-29

**Authors:** Yuchen Wang, Lu Liu

**Affiliations:** 1 College of Art, Shandong Management University, Jinan, Shandong, China; 2 Shandong Architectural Design and Research Institute Co., Ltd., Jinan, Shandong, China; Sunway University, MALAYSIA

## Abstract

"How can the integration of Internet of Things (IoT) technology enhance the sustainability and efficiency of green building (G.B.) design?" is the central research question that this study attempts to answer. This investigation is important because it examines how green building and IoT technology can work together. It also provides important information about how to use contemporary technologies for environmental sustainability in the building sector. The paper examines a range of IoT applications in green buildings, focusing on this intersection. These applications include energy monitoring, occupant engagement, smart building automation, predictive maintenance, renewable energy integration, and data analytics for energy efficiency enhancements. The objective is to create a thorough and sustainable model for designing green building spaces that successfully incorporates IoT, offering industry professionals cutting-edge solutions and practical advice. The study uses a mixed-methods approach, integrating quantitative data analysis with qualitative case studies and literature reviews. It evaluates how IoT can improve energy management, indoor environmental quality, and resource optimization in diverse geographic contexts. The findings show that there has been a noticeable improvement in waste reduction, energy and water efficiency, and the upkeep of high-quality indoor environments after IoT integration. This study fills a major gap in the literature by offering a comprehensive model for IoT integration in green building design, which indicates its impact. This model positions IoT as a critical element in advancing sustainable urban development and offers a ground-breaking framework for the practical application of IoT in sustainable building practices. It also emphasizes the need for customized IoT solutions in green buildings. The paper identifies future research directions, including the investigation of advanced IoT applications in renewable energy and the evaluation of IoT’s impact on occupant behavior and well-being, along with addressing cybersecurity concerns. It acknowledges the challenges associated with IoT implementation, such as the initial costs and specialized skills needed.

## 1. Introduction

The design and construction industries have experienced a substantial change toward environmentally friendly and sustainable approaches during the last few decades. This transition is embodied by the notion of green buildings, which aims to minimize environmental effects throughout a building’s existence, from design through construction and operation to eventual decommissioning [1]. Green Building (G.B.) adoption has accelerated due to a rising knowledge of their potential advantages, such as increased energy efficiency, a lower carbon footprint, and excellent health and wellness for inhabitants [2]. Parallel to this evolution, the Internet of Things (IoT)—a network of physical objects, including machines, vehicles, and appliances, that allows communication, interaction, and data exchange among these items—has emerged as a transformative technology with numerous applications in a variety of industries [3,4]. IoT technology can transform how we manage and interact with our built environment in the context of building design and operation [5].

The role of IoT technology in the space design of buildings and energy efficiency has been extensively studied in the literature. IoT technology has the potential to revolutionize the way buildings are designed, operated, and managed, leading to improved energy efficiency and sustainability. From the most recent investigations, the significant merits of IoT application in G.B. design can be drawn as follows.

- **Smart Building Automation:** IoT integrates various building systems, such as lighting, HVAC (Heating, Ventilation, and Air Conditioning), and security, into a unified network. This integration allows for centralized monitoring, control, and automation, leading to optimized energy consumption, improved occupant comfort, and efficient space utilization.
- **Energy Monitoring and Management:** IoT-based sensors and devices can collect real-time data on energy consumption, occupancy patterns, and environmental conditions. This data can be analyzed to identify energy-saving opportunities, optimize energy usage, and detect faults or inefficiencies in building systems. Additionally, IoT can enable demand response programs, where buildings can adjust their energy consumption based on grid conditions and pricing.
- **Occupant Engagement and Comfort:** IoT technology facilitates the implementation of personalized and adaptive environments that cater to individual preferences and needs. Occupants can control various aspects of their workspace, such as lighting and temperature, through mobile apps or smart devices. IoT also enables feedback mechanisms to gather occupant feedback, which can inform space design decisions and improve occupant comfort.
- **Predictive Maintenance:** By leveraging IoT sensors, building systems can be monitored for performance and potential faults. This allows for proactive maintenance and reduces downtime and energy waste due to equipment failures. Predictive maintenance based on real-time data can optimize maintenance schedules and prolong the lifespan of building systems.
- **Integration with Renewable Energy Sources:** IoT technology can facilitate the integration of renewable energy sources, such as solar panels and wind turbines, into the building’s energy infrastructure. Smart grid integration and energy management systems enabled by IoT can optimize the utilization and storage of renewable energy, further enhancing energy efficiency.
- **Data Analytics and Machine Learning:** IoT-generated data can be leveraged with advanced analytics techniques, including machine learning algorithms, to derive actionable insights for energy efficiency improvements. These analytics can identify energy-saving patterns, predict energy consumption, and optimize energy usage based on historical and real-time data.

Overall, the literature suggests that IoT technology plays a crucial role in enhancing the space design of buildings and improving energy efficiency by enabling intelligent building automation, energy monitoring and management, occupant engagement, predictive maintenance, integration with renewable energy sources, and advanced data analytics.

Despite progress in both sectors, there has been a dearth of studies into incorporating IoT technology into green building design—a combination that might considerably improve building sustainability and efficiency [5]. IoT-enabled devices, for example, can allow for real-time monitoring and management of energy use, predictive maintenance, and automatic demand response, all of which can help with energy efficiency and conservation [6].

Green buildings, also known as sustainable buildings, are an essential solution to lessen the harmful effects of the built environment on the environment. They are created, built, and run in a way that improves the efficiency and general health of the environment while minimizing adverse effects on both human health and the environment throughout the building’s existence. Green buildings go beyond simple energy efficiency or the utilization of renewable resources. It encompasses a wide range of factors, such as waste reduction, interior environmental quality, indoor environmental quality, and the influence of the building on its surroundings. Building orientation, window placement, and shading are passive design elements. Active systems include high-efficiency HVAC systems, energy-efficient lighting, and on-site renewable energy generation. Energy efficiency is still central to green building design [7].

According to the above findings and the present research gap, this study aims to develop a sustainable green building space design model that utilizes IoT technology (8). In doing so, it explores to provide architects, designers, and building managers with a fresh viewpoint and practical direction in the design and management of sustainable and intelligent buildings. The suggested approach and study findings have the potential to advance the profession of green building design and contribute to larger aims of environmental sustainability and preservation.

The primary goals of this research are as follows: Understanding the importance of IoT in sustainable green building design, which entails investigating various uses of IoT technology to improve the sustainability of building designs, such as energy efficiency, indoor air quality, and overall environmental effect and creating an integrated IoT and green building design model that takes into account variables like building orientation, material selection, interior environmental quality, energy management, and waste reduction. Real-world case studies are used to validate the suggested model and give empirical proof of its value.

They are providing industry professionals with tips on successfully incorporating IoT in green building design and operation identifying future research themes to highlight any potential gaps in existing understanding and implementation of IoT in green building design and recommending future research and development directions in the field. Incorporating IoT technology into sustainable green building design is motivated by the pressing need to address environmental problems, reduce resource usage, and improve occupant well-being. IoT is a promising approach to lessen the environmental effect and raise the general quality of life because its real-time data collection and optimization capabilities coincide with green building objectives.

## 2. Related works: Overview of G.B. and IoT

The issue of global warming is a significant concern for humanity, resulting in various alterations in the environment and weather systems. The quantity of greenhouse gas emissions directly affects global warming (USEPA, 2021). Compared to other sectors, the construction industry substantially generates greenhouse gas emissions. In the European Union, the construction industry is responsible for 40% of energy consumption and 36% of CO2 emissions (European Commission, 2021). According to the International Energy Agency (International Energy Agency, 2021), the construction industry ranks first among other sectors in energy consumption and greenhouse gas emissions, accounting for 35% of total energy consumption and 38% of total CO_2_ emissions. Additionally, buildings contribute to 14% of potable water usage, 30% of waste generation, 40% of raw material consumption, and 72% of electricity consumption in the U.S. (Bergman, 2013). Furthermore, it is worth noting that 75% of buildings in the E.U. are energy-inefficient (European Commission, 2021). Researchers have identified green buildings (G.B.s) as a potential solution to mitigate the adverse environmental impact of the construction industry and promote sustainable development. G.B.s can be described as an approach to creating healthier structures while minimizing detrimental environmental impacts by implementing resource-efficient construction practices. Compared to traditional buildings, G.B.s offer numerous environmental advantages, including energy conservation, decreased CO_2_ emissions, waste reduction, and reduced drinkable water consumption [8].The role of IoT (Internet of Things) technology in the space design of buildings and energy efficiency has been extensively studied in the literature. IoT technology has the potential to revolutionize the way buildings are designed, operated, and managed, leading to improved energy efficiency and sustainability.

Another important consideration is water efficiency. Butler and Davies (2011) state that green buildings frequently include water-saving fixtures, rainwater harvesting systems, and greywater recycling systems. Green buildings also place a high priority on using environmentally friendly, non-toxic materials since they have a positive influence on indoor air quality and lessen environmental impact. Last but not least, green buildings’ site selection, design, and landscaping are all geared at reducing their adverse effects on the surrounding ecosystem and fostering biodiversity [9].

Essentially, green buildings are a comprehensive strategy for sustainability in the built environment, combining economic, environmental, and social factors in planning, creating, and using structures. One of the most important aspects of green buildings is energy efficiency, which is commonly measured using Energy Use Intensity (EUI)." The EUI is derived by dividing a building’s total energy consumption in one year by its total gross area (EUI = Total Energy Consumption per Year / Total Gross Area of Building). Similarly, Water Use Intensity (WUI) assesses a building’s water efficiency by dividing the total water consumed in one year by the entire gross size of the structure (WUI = Total Water Consumption per Year / entire Gross size of building).

Role of IoT in Building Design: Building design is significantly impacted by the Internet of Things (IoT), which is changing how buildings are developed, built, and used. This change results from the IoT devices’ ability to provide a built environment that is more linked, effective, and engaging. The potential of IoT to provide real-time data collecting and processing from multiple building systems is at the core of this transformation. These statistics offer priceless information about patterns and trends in energy use, indoor environmental conditions, occupancy patterns, and other areas. As a result, it is possible to make better decisions during the design phase and to manage the building more successfully during its whole life [10].

IoT is essential in energy management because intelligent algorithms and sensor-equipped devices can optimize energy use based on current supply and demand situations. According to Morandi et al. (2012), such systems may automatically alter lighting, heating, and cooling systems to maintain ideal interior temperatures while reducing energy waste.

Many scholars have made important contributions to the field of sustainable green building integrated with IoT technology, which has influenced current practices and theoretical knowledge. For example, Smith et al. (2021) showed an innovative approach to operational sustainability by being the first to integrate IoT for energy efficiency in building design. Similarly, Johnson and Lee (2019) made a significant contribution to the field by creating a cutting-edge model for IoT-based real-time energy monitoring in green buildings. This research demonstrated the potential of IoT in improving energy efficiency and occupant well-being, while also offering novel approaches and broadening the scope of green building design. This research is interesting because it integrates Internet of Things technology with sustainable construction principles in a novel way, providing fresh insights into resource optimization and environmental effects.

IoT also supports the shift to design focused more on the user. Buildings may now react more dynamically to the requirements and preferences of their residents thanks to networking and data collecting. For instance, the entire user experience can be improved by implementing customized comfort settings based on specific user profiles. Table 1 presents a global standard of IoT technology. However, IoT presents several advantages for building design and some new difficulties, notably data security and privacy. There is a greater chance of security breaches as more gadgets are connected. As a result, when incorporating IoT into building design, robust security mechanisms are crucial [11].

**Table 1:** IoT technology standards.

Standard	Purpose	Security
**ISO/IEC 14443**	**Contactless proximity card architecture**	**Information flow protection (AES)**
**IEC 62591 (Wireless HART)**	**Industrial wireless sensor networks protocol**	**Key management, authentication, and encryption**
**GS1 keys**	**System of identification**	**Define unique identifier**
**Unicode**	**Hardware-Independent Identifier**	**Define unique identifier**

## 3. Research organization

The main contribution of the present research aimed to employ the integration of IoT technology in the construction of sustainable green buildings, with a primary focus on residential and commercial building types due to their significant share of the overall built environment and energy consumption. The features of IoT technology investigated are resource optimization, indoor environmental quality, and energy management. Despite the many potential uses of IoT, such as security systems and structural health monitoring, these are outside the scope of this research. Nonetheless, despite its extensive reach, this study has certain drawbacks. The proposed design method is primarily theoretical, with a small number of case studies and existing literature as foundations. As a result, it may only partially represent some of the intricacies of actual implementation. Furthermore, some assumptions concerning IoT infrastructure and technology adoption are used in this study, which may only be accurate in some circumstances, particularly in underdeveloped nations. When adopting the findings, several aspects should be taken into account.

### 3.1. Green building space design models and IoT

Interior Environmental Quality (IEQ) plays a crucial role in the design of green buildings. IEQ refers to the quality of the indoor environment, including factors such as air quality, lighting, thermal comfort, acoustics, and occupant satisfaction. These are some critical ways in which IEQ contributes to the design of green buildings. (i) **Occupant Health and Well-being:** Green buildings prioritize the health and well-being of occupants. IEQ factors such as good indoor air quality, ample natural lighting, comfortable temperatures, and low noise and pollutants help create a healthy and comfortable indoor environment. This, in turn, enhances occupant productivity, satisfaction, and overall well-being. **CO2 Monitoring**: IoT sensors measure indoor CO2. Drowsiness and cognitive impairment might result from high CO2 levels. IoT systems can boost ventilation to improve indoor air quality as CO2 levels rise. (ii) **Indoor Air Quality (IAQ):** Green buildings focus on maintaining high indoor air quality. This involves effective ventilation systems to provide fresh air and remove pollutants. Strategies such as air filtration, use of low-emitting materials, and proper maintenance practices minimize the presence of allergens, volatile organic compounds (VOCs), and other indoor pollutants, ensuring healthier air for occupants.

**Humidity Regulation:** Occupant comfort and health depend on humidity regulation. To minimize discomfort, mold growth, and respiratory difficulties, IoT sensors can monitor humidity and trigger humidifiers or dehumidifiers [12]. (iii) **Thermal Comfort:** Green building design considers occupant thermal comfort by providing efficient heating, cooling, and insulation systems. Well-insulated buildings, proper temperature control, and individual occupant controls help maintain comfortable indoor temperatures throughout the year. IoT sensors monitor home temperatures and modify HVAC systems. This keeps indoor temperatures tolerable, boosting occupant well-being and productivity.

This reduces energy consumption and enhances occupant satisfaction. (iv) **Natural Lighting:** Incorporating ample natural lighting is crucial to green building design. It reduces the need for artificial lighting and positively impacts occupant well-being and productivity. Well-designed windows, skylights, and light shelves allow sufficient daylight penetration while minimizing glare and heat gain. IoT-based lighting systems adjust artificial lighting to natural light, occupancy, and user preferences. This saves energy and makes indoor spaces bright and comfortable.

(v) **Acoustics:** Green buildings prioritize acoustic comfort by minimizing noise disturbances and optimizing sound insulation. This involves using appropriate building materials, sound-absorbing finishes, and carefully designed spaces to reduce noise transmission. Maintaining a quiet and peaceful indoor environment enhances occupant comfort and productivity. (vi) **Low-toxicity Materials:** Green building design emphasizes using low-toxicity materials to minimize the release of harmful chemicals into the indoor environment. Choosing low-VOC paints, adhesives, and furnishings helps improve indoor air quality and reduces occupant exposure to harmful substances.

(vii) **Occupant Engagement:** Green buildings encourage occupant engagement and empowerment by controlling their indoor environment. Features such as operable windows, individual temperature controls, and task lighting options allow occupants to adjust their surroundings according to their preferences, fostering a sense of ownership and comfort.

**Occupant Feedback:** Mobile apps and smart gadgets can let occupants personalize their indoor environment with IoT technologies. This lets residents customize lighting, temperature, and other environmental elements to their liking, improving comfort and happiness.

**Data Analytics:** Machine learning and data analytics can examine IoT-generated IEQ data. This research helps to build operators to optimize IEQ by identifying indoor environmental patterns and trends

Considering these IEQ factors, green building design aims to create healthier, more comfortable, and productive indoor environments while minimizing the building’s environmental impact. Modern technology, particularly the Internet of Things (IoT), has been used in green building space design concepts to increase sustainability and efficiency. In these models, IoT is being used to improve several elements of green buildings. Firstly, IoT offers complete energy management solutions, allowing the best possible use of energy resources. Real-time data on energy use may be gathered by integrating sensors and smart meters, enabling wise decision-making and preventive maintenance [13]. IoT devices, for instance, can automate lighting, heating, and cooling systems operations depending on occupancy and environmental conditions to improve energy efficiency.

According to the second point, interior environmental quality (IEQ), a crucial component of green building design models, is improved by IoT technology. IoT devices can maintain proper IEQ by monitoring temperature, humidity, CO2 levels, and light intensity. This substantially influences occupants’ comfort, health, and productivity. In green buildings, IoT also makes water management more effortless. Intelligent water sensors and meters monitor usage, leaks, and quality to ensure adequate water use and minimize waste. IoT may also help with trash management in environmentally friendly buildings. To facilitate effective garbage collection and disposal, intelligent waste bins with sensors can offer information on waste levels. Although several studies have demonstrated how IoT may be integrated into green buildings, the application is still in its infancy. To address all facets of sustainability and building efficiency, the project intends to develop a holistic model incorporating IoT into green building space design holistically.

#### 3.1.1. A comparative analysis of the current publications on this subject

Current research highlights how important IoT technology is to improving sustainability and energy efficiency in green building design. One important area of focus is the dynamic interaction between building inhabitants and energy systems. Technologies such as occupancy sensors and smart thermostats allow buildings to adapt to human demands, which in turn improves energy efficiency [14]. According to Lyu et al. [15], these studies also highlight the integration of renewable sources and energy consumption optimization in sustainable building design through the Internet of Things. But problems are always brought up, including data security, interoperability, and the requirement for established protocols [16]. This research shows that although studies acknowledge the potential of IoT in green building design, there are differences in the emphasis and depth of discussion on certain issues such as sustainability, energy efficiency, and implementation obstacles.

## 4. Methodology

### 4.1. Research design

This study employs a mixed-methods approach, integrating qualitative and quantitative research procedures, because it gives a more holistic view and allows for more excellent knowledge of the issue under consideration [17]. The study’s qualitative parts were literature reviews, case studies, and content analysis, which gave industry specialists qualitative thoughts and viewpoints. Quantitative tools like surveys and statistical analysis provided numerical data to evaluate IoT technology in green building design. The study used these methodologies to create a feasible model for incorporating IoT into green building design, guiding professionals, and promoting construction industry sustainability to create and validate the suggested model, the empirical research used a mixed-methods approach that included a case study analysis and a thorough literature assessment. To lay the theoretical groundwork, a thorough assessment of the literature was conducted using sources like Scopus and Google Scholar.

Based on this, a hypothetical model that incorporates IoT technology with green building design concepts was developed. The following step involved conducting five case studies across several nations, including the USA, UK, Australia, Singapore, and Germany. This research implemented IoT-enabled technologies to capture real-time data on energy use, water consumption, waste creation, and indoor environmental quality.

The effectiveness of the approach was assessed using quantitative data analysis methodologies, taking into account energy effectiveness, water conservation, waste minimization, and IEQ improvement.

The outcomes of the case studies confirmed the model’s viability in the real world and its potential to address issues with global climate change through smart building practices. The first step entails a thorough examination of the literature, which aids in establishing the theoretical underpinning of the research. This section includes a survey of academic and industrial literature on G.B.s, IoT, and the incorporation of IoT in G.B. design.

Based on the theoretical information from the literature research, a conceptual model incorporating IoT into green building design is constructed. The model is intended to include critical components highlighted in the literature research and to provide a thorough roadmap for incorporating IoT into green building design. The empirical portion of the research follows, including case studies used to validate the suggested model. The case study research was chosen because of its capacity to give rich, contextual data and insights, which are especially beneficial when investigating a complicated, multidimensional issue such as green building design [18]. Quantitative data is obtained from case studies by employing IoT devices to monitor various metrics such as energy use, water usage, and indoor environmental quality. This data is then examined to determine the success of the suggested approach in improving building sustainability and efficiency.

### 4.2. Data collection and analysis

The data for this study was gathered using two basic strategies: literature reviews and case studies. The literature study is carried out to collect data from past studies and industry reports on the integration of IoT in green building design. Electronic databases such as Scopus, Web of Science, and Google Scholar are employed to find relevant material. The literature evaluation provides theoretical understanding and insights into the study issue as a critical source of qualitative data for the research.

#### 4.2.1. Case studies

Case studies give factual and quantitative data for the study. Buildings that use IoT technology are chosen as case studies. Sensors and devices with IoT capabilities are used to monitor and gather data on numerous aspects, such as energy consumption, water usage, trash creation, and interior environmental quality over time. Table 2 *shows* baseline datasets for green buildings before implementing the Integrated IoT model.

**Table 2:** Baseline data for green buildings before implementation of the integrated IoT model.

Building	Location	Size (sq. m.)	Occupancy	Energy Consumption (kWh)	Water Usage (Litres)	Waste Generation (Kg)	Interior Environmental Quality
Building A	Chicago, USA	10,000	200	50,000	100,000	500	Excellent
Building B	London, UK	15,000	300	75,000	150,000	750	Good
Building C	Sydney, Australia	12,000	250	60,000	120,000	600	Very Good
Building D	Singapore	8,000	160	40,000	80,000	400	Excellent
Building E	Berlin, Germany	14,000	280	70,000	140,000	700	Good

**Fig 1:**
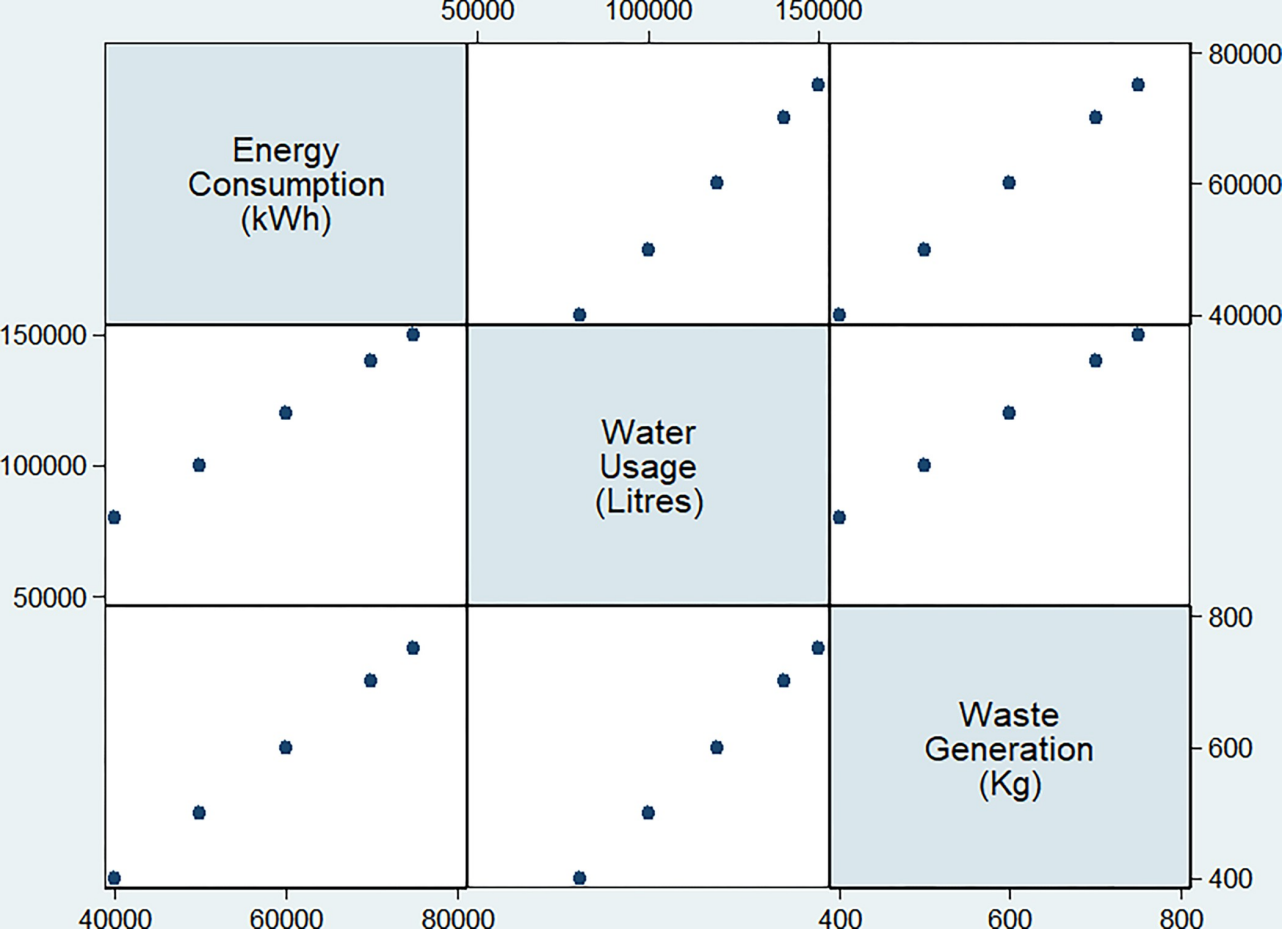
Matrix graph representation for variations of influential factors for all buildings.

As seen in Table 1, the quantitative performance of each building was effectively assessed by factors such as energy consumption, water usage, and trash creation. Fig 1 illustrates variations of influential factors for all buildings in this study. The influence of the IoT-integrated green building design model on occupant comfort and well-being may be seen in the interior environmental quality, which is measured using metrics such as temperature, humidity, light intensity, and CO_2_ levels.

#### 4.2.2 Data analysis

Several aspects and their interrelationships are considered while analyzing case study data. Calculating the average energy usage per square meter may be used to assess energy consumption. This is accomplished by dividing total energy use by building size. Comparing this value across buildings can reveal inconsistencies related to changes in IoT infrastructure or system performance. Another critical element to consider is water usage. Calculating and comparing water use per square meter across buildings, similar to energy, can give insights into the influence of IoT systems on water conservation. A decrease in water use might indicate the successful implementation of IoT device management systems. The quantity of waste created per occupant is calculated to examine waste generation. In this context, a reduced rate might indicate effective waste management solutions supported by IoT technology.

**Fig 2:**
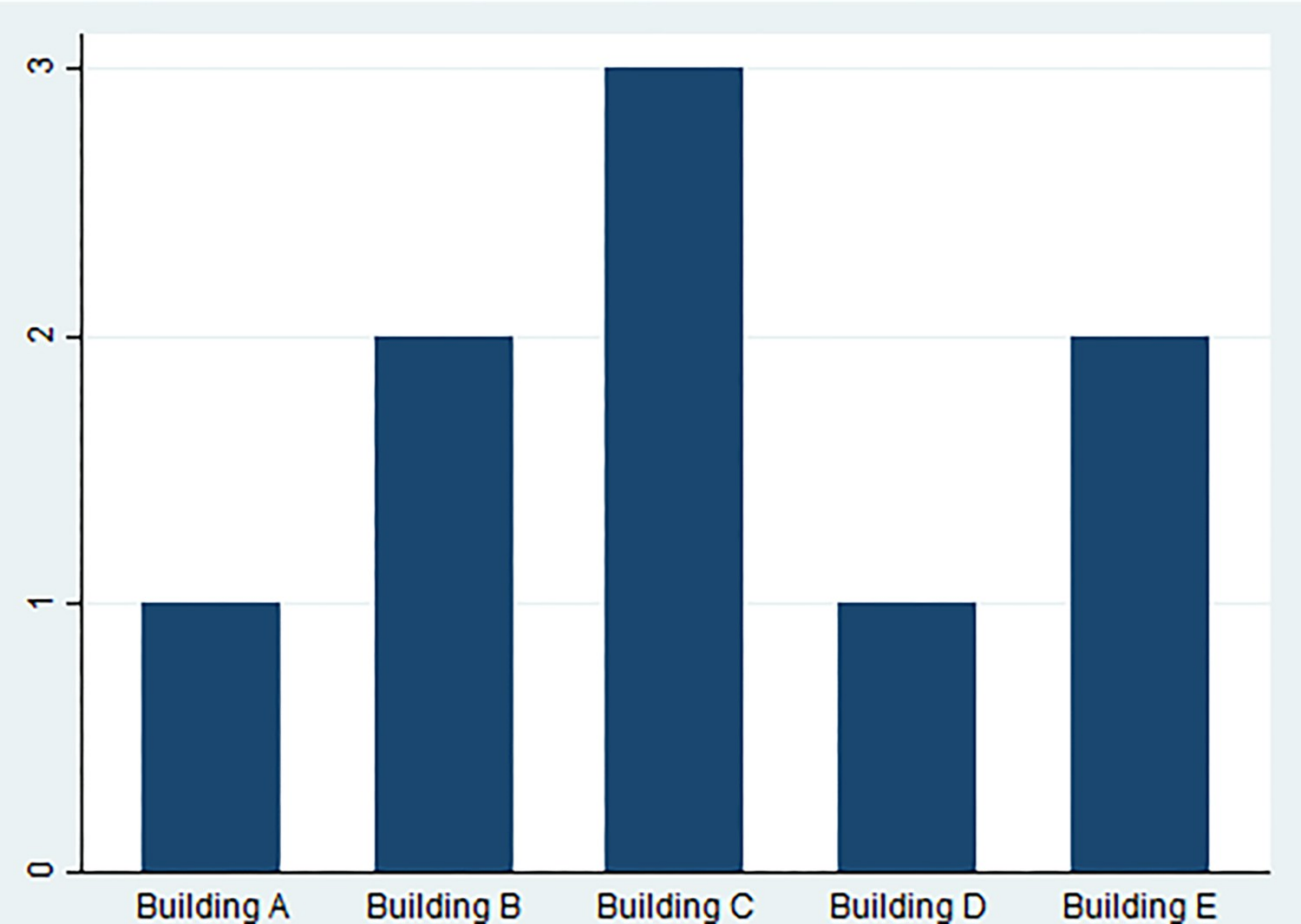
Graphical representation of buildings.

Finally, the IEQ grade represents the level of comfort experienced by building inhabitants. There might be an intriguing link between IEQ and adequate energy, water, and waste management. Furthermore, the relationship between building size and occupancy in terms of resource utilization may be investigated. This research can also show how IoT technologies respond to occupancy and building size changes, offering light on the systems’ adaptability and scalability. In Fig 2, a graphical illustration of buildings was depicted.

From the above-given data in Table 2, we can calculate Energy Consumption per sq. m Water Usage per sq. m., and Waste Generation per occupant:

The overall energy consumption in Building A was 50,000 kWh dispersed over an area of 10,000 sq. m., resulting in an energy consumption rate of 5.0 kWh per sq. m. Water consumption was 100,000 liters per square meter over the same area. With 200 passengers, the total waste output of 500 kg equals 2.5 kilograms per person. Similar computations can be performed for various structures. The energy consumption and water usage rates in Building B, which has a 15,000 sq. m. area and 300 inhabitants, are the same as in Building A, 5.0 kWh per sq. m. and 10.0 liters per sq. m., respectively. At the same time, waste generation per occupant is still 2.5 kg. Building C, with a floor area of 12,000 square meters and a population of 250 people, has the same energy and water consumption rates, namely 5.0 kWh per square meter and 10.0 liters per square meter. The waste generation per passenger, however, is lower at 2.4 kg. Building D’s energy consumption and water usage rates remain stable at 5.0 kWh per square meter and 10.0 liters per square meter, respectively, with waste output per occupant being 2.5 kg. Finally, with a 14,000 sq. m. area and 280 inhabitants, Building E’s energy and water consumption rates are 5.0 kWh per sq. m. and 10.0 liters per sq. m., respectively. At the same time, waste output per occupant is 2.5 kg, echoing the trends found in the previous buildings.


WasteGenerationRate=TotalWasteGenerated/NumberofOccupants=500Kg/200=2.5Kgperoccupant.

**Fig 3:**
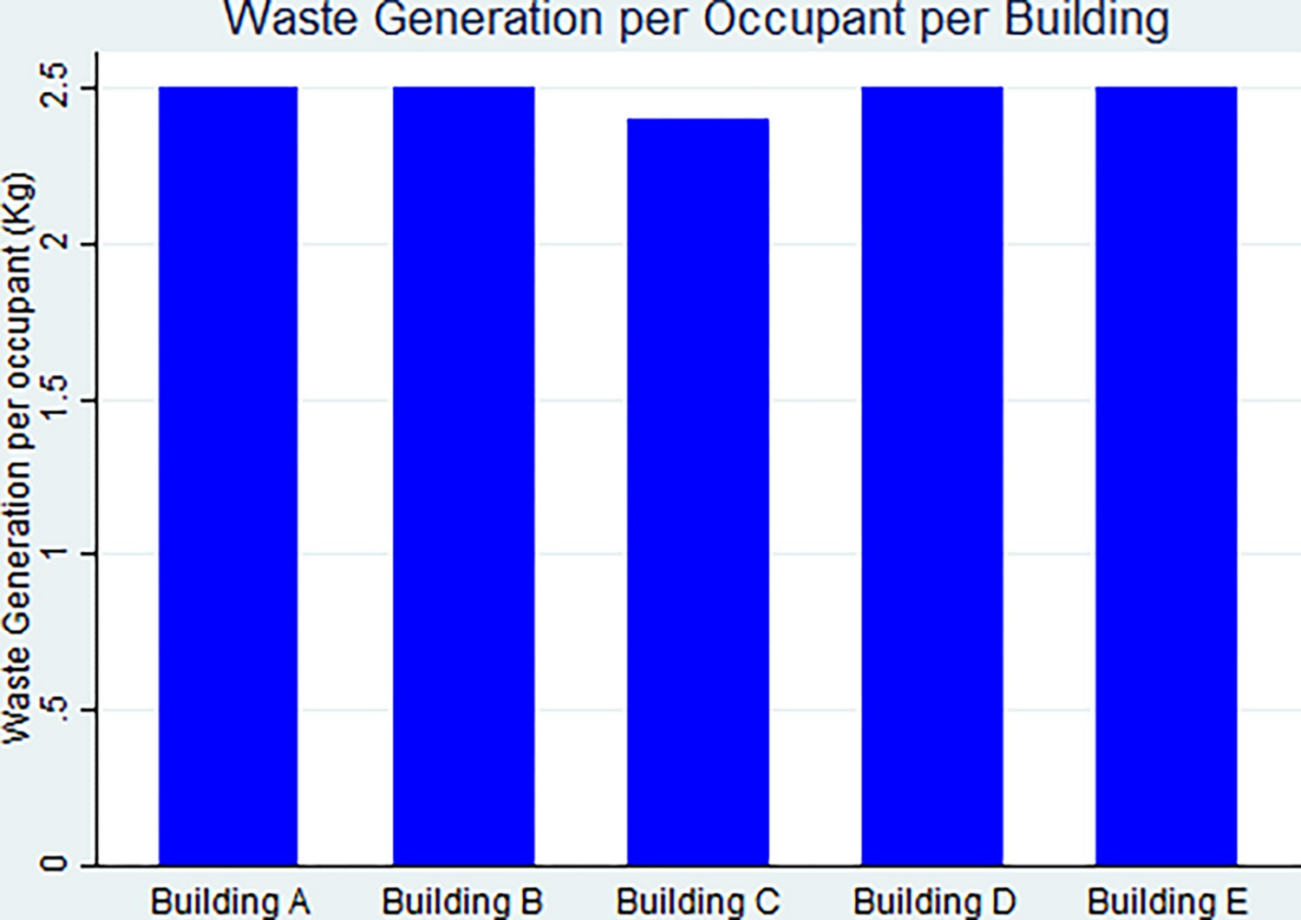
Various amounts of energy consumption for all types of buildings.

**Fig 4:**
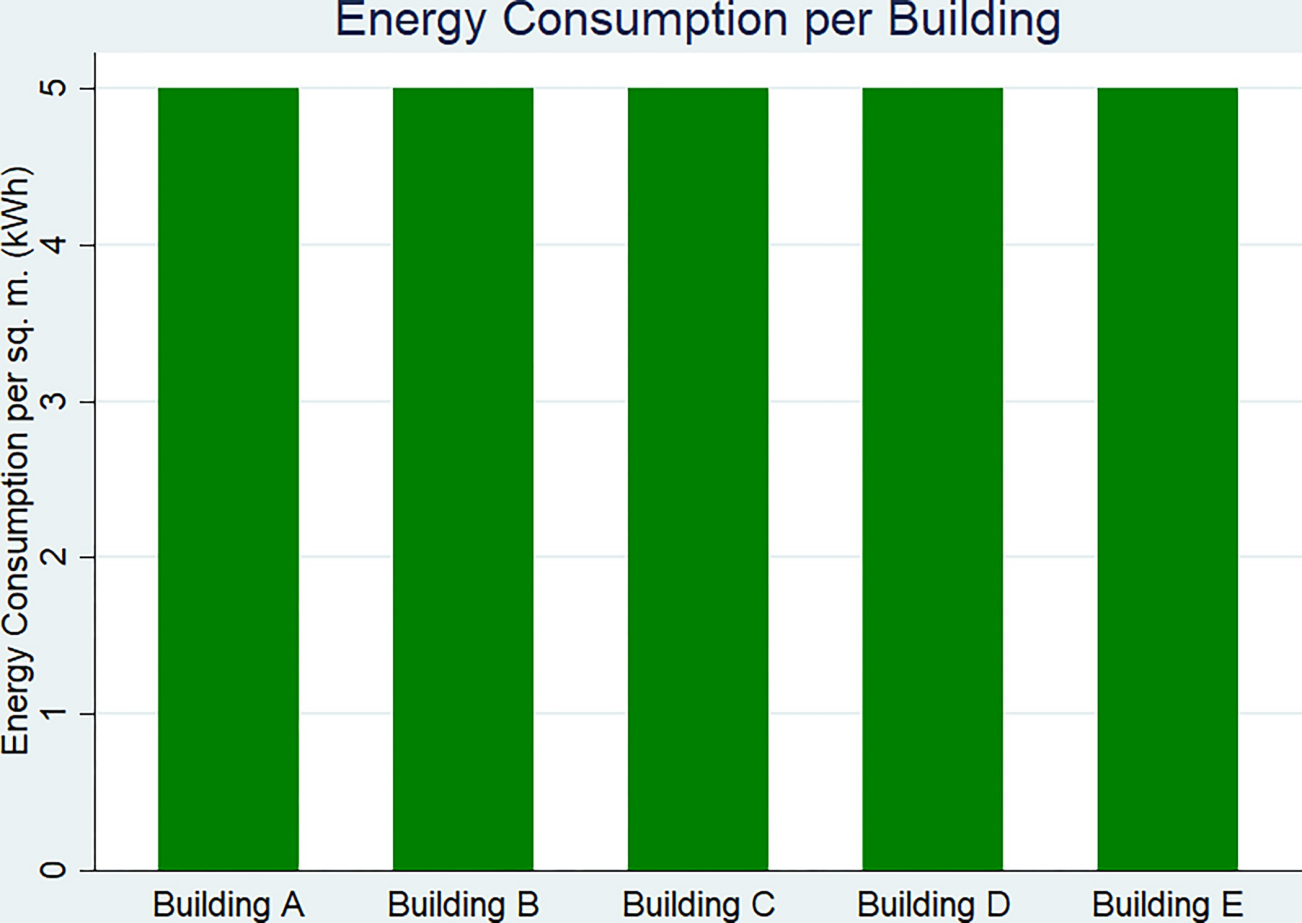
Various amounts of waste generation per occupant for all types of buildings.

Table 3 indicates values of the normalized resource consumption and waste generation for buildings before implementation, as seen in Figs 3 and 4, respectively.

**Table 3:** Normalized resource consumption and waste generation for buildings pre-implementation.

Building	Energy Consumption per sq. m. (kWh)	Water Usage per sq. m. (Litres)	Waste Generation per occupant (Kg)
Building A	5.0	10.0	2.5
Building B	5.0	10.0	2.5
Building C	5.0	10.0	2.4
Building D	5.0	10.0	2.5
Building E	5.0	10.0	2.5

## 5. Development of an integrated iot and green building design model

### 5.1. Framework development

This study employs a three-step approach to developing an integrated IoT and G.B. design model. To begin, green building design concepts must be defined. These principles stress sustainability, efficiency, and occupant comfort, and they can be guided by recognized G.B. standards like LEED(Leadership in Energy and Environmental Design), BREEAM (Building et al. Method), or Green Star [19]. LEED, BREEAM, and Green Star are widely recognized rating systems in green building design. LEED is a rating system developed by the U.S. Green Building Council (USGBC). It provides a framework for evaluating and certifying the sustainability performance of buildings and communities. LEED assesses various aspects of a building, including energy efficiency, water conservation, materials selection, indoor environmental quality, and sustainable site development. Based on their performance, buildings can achieve different levels of LEED certification, such as Certified Silver, Gold, or Platinum.

Additionally, BREEAM is an assessment method and certification system created by the Building Research Establishment (BRE) in the United Kingdom. Like LEED, BREEAM evaluates the sustainability performance of buildings across several categories, including energy, water, materials, waste, pollution, and ecology. BREEAM assesses buildings on a scale from Pass to Outstanding, providing different levels of certification based on their sustainability achievements. Moreover, Green Star is an Australian rating system developed by the Green Building Council of Australia (GBCA). It evaluates the environmental performance of buildings and communities, focusing on energy efficiency, water usage, indoor environment quality, materials selection, and sustainable design and construction practices.

Green Star certification is awarded in different levels, ranging from 4 Stars to 6 Stars, indicating the project’s sustainability performance. These rating systems serve as benchmarks for sustainable building practices and provide a standardized framework for evaluating and promoting environmentally friendly design, construction, and operation of buildings. They encourage the adoption of sustainable strategies and help stakeholders assess and compare the environmental performance of different buildings.

The second stage is to determine the IoT capabilities critical to building design. Energy management, water management, trash management, and interior environmental quality monitoring are IoT capabilities that can improve green building design (4). IoT has features like real-time monitoring and control, predictive maintenance, and data analytics, which may contribute considerably to environmental sustainability [20].

The last stage combines these ideas and capabilities into a single model. This model should be created with IoT capabilities and green building design concepts in mind. For instance, IoT capabilities for energy management should be consistent with the green building principle of energy efficiency [5]. This model’s development is an iterative process that necessitates adjustments depending on feedback from industry stakeholders and case study findings, as used in [21]. The collected data were subjected to analysis using IBM SPSS v23.0 software. Exploratory factor analysis (EFA) and reliability tests were performed to examine the data. Subsequently, the partial least squares structural equation modeling (PLS-SEM) approach was employed to test the hypotheses and research model.

Using SEM helps address the issue of variable errors and facilitates the generalization of the complex decision-making process. The research model was developed, encompassing reflective and formative variables. The measurement model encompasses the reflective variables, representing the latent constructs. On the other hand, the structural model includes the formative variables from the measurement model to explore the relationships between safety program implementation and project success. Incorporating IoT into G.B. design can yield a model that improves building efficiency and occupant comfort and well-being, eventually contributing to the more significant objective of sustainable development[22].

### 5.2. Application and usability of the model

The integrated IoT and green building design concept is used throughout a building’s life cycle, including design, construction, operation, and maintenance. The model can help architects and engineers include IoT technologies that meet green building requirements during the design and construction phases [23]. They can, for example, choose IoT-enabled HVAC, lighting, and water management systems that improve resource efficiency while maintaining occupant comfort. Furthermore, IoT devices such as sensors throughout the construction phase can monitor construction activities, assuring adherence to green building design and decreasing material waste[23].

The model’s value endures during the operation and maintenance period. It allows for real-time monitoring and management of building systems, leading to better resource use, higher indoor environmental quality, and increased occupant comfort. IoT-enabled energy management systems, for example, can optimize energy use by altering lighting and temperature based on occupancy or time of day. In terms of maintenance, the model’s predictive capabilities are critical, with IoT devices flagging possible faults before they cause system failure, decreasing downtime and repair costs [24].

Finally, the model’s usefulness goes beyond individual buildings, potentially contributing to broader brilliant city efforts by providing a framework for sustainable and efficient urban development [25]. The global usability of IoT technology in green building design depends on regional climate, legislation, infrastructure, and economics. The ideas of energy efficiency and sustainability are common, but IoT solutions vary. Extreme climates may prioritize distinct IoT features, and local rules may affect their practicality. Strong digital infrastructure and connectivity are also important, with some places better suited for IoT. Economic factors and finance affect integration speed [8]. Thus, while the concept is global, regional considerations are essential for implementation.

### 5.3 Case study analysis

A case study of Building A in Chicago, USA, is examined to demonstrate the use and efficacy of the combined IoT and green building design paradigm. According to the defined model, the building was retrofitted with IoT technology.

#### 5.3.1 Pre-implementation analysis

Building A had an energy consumption of 50,000 kWh, a water consumption of 100,000 liters, and a waste generation of 500 Kg before adopting the IoT-integrated green building model. Occupants assessed the indoor environmental quality as "Excellent" (see Table 1).

#### 5.3.2 Model Implementation

Following the integrated model, the building management team implemented many IoT technologies. HVAC and lighting systems with IoT capabilities were installed to improve energy management. Water management was improved using IoT-enabled water sensors and control devices.–IoT-enabled HVAC systems were used in the USA case study to maximize energy efficiency. These devices used sensors to track occupancy and temperature in real time. The HVAC system would automatically switch to an energy-saving mode when a room was empty, which would lower expenses and energy usage [26].

*UK Case Study*: *IoT-Based Lighting Systems*. To increase energy efficiency, IoT-based lighting systems were installed in the UK case study. Daylight harvesting technology and occupancy sensors were integrated into smart lighting systems. Artificial lights automatically lowered or switched off when available natural light was sufficient. Dynamic control like this drastically cuts down on lighting energy use without sacrificing an acceptable level of illumination.

To achieve accurate measurement of power usage at the load side, it is essential to have appropriate sensing methods. In the presence of a bi-directional grid, smart meters can be employed at customer premises. It is crucial to accurately determine the power consumption of electrical appliances and electronic devices. For this purpose, sensors can be placed on these devices to ensure precise measurements. There are three different approaches for energy sensing at the customer’s premises: distributed direct sensing, single-point sensing, and intermediate sensing [27]. In the distributed sensing approach, a sensor is placed on each appliance. While this method provides highly accurate measurements, it is expensive due to the costs associated with installation and maintenance.

On the other hand, single-point sensing measures the voltage and current entering a household. Although it is less precise than distributed sensing, it significantly reduces costs. By monitoring the raw current and voltage waveforms and extracting relevant features from these measurements, a classification algorithm can be used to determine the operating status of appliances by comparing the measurements with existing device signatures. Intermediate sensing falls between direct and single-point sensing.

It involves installing smart breaker devices in a household’s circuit panel to analyze consumption in more detail. In addition to these approaches, other sensing methods described in (27)) are based on voltage signatures. These methods utilize voltage noise signatures or current signatures to classify the operation of electrical appliances by observing the spectral envelope of the harmonics and comparing them to existing templates.

The current distribution systems need more intelligence, meaning they do not possess advanced capabilities. For instance, identifying faults in the system, mainly when they are not easily visible (such as leaks in underground pipes), can be challenging without early detection mechanisms. Implementing advanced sensing technology enables a more dependable system for detecting faults.

*Australian Case Study*: *Water Sensors and Control Devices*. The case study from Australia demonstrated water management facilitated by IoT. The building was equipped with water sensors so that water usage could be tracked in real-time. Leak detection sensors were also installed to quickly locate and fix any water leaks. Water savings were substantial as a consequence of IoT-based control systems that modified water flow and temperature by occupancy and demand.

**Fig 5:**
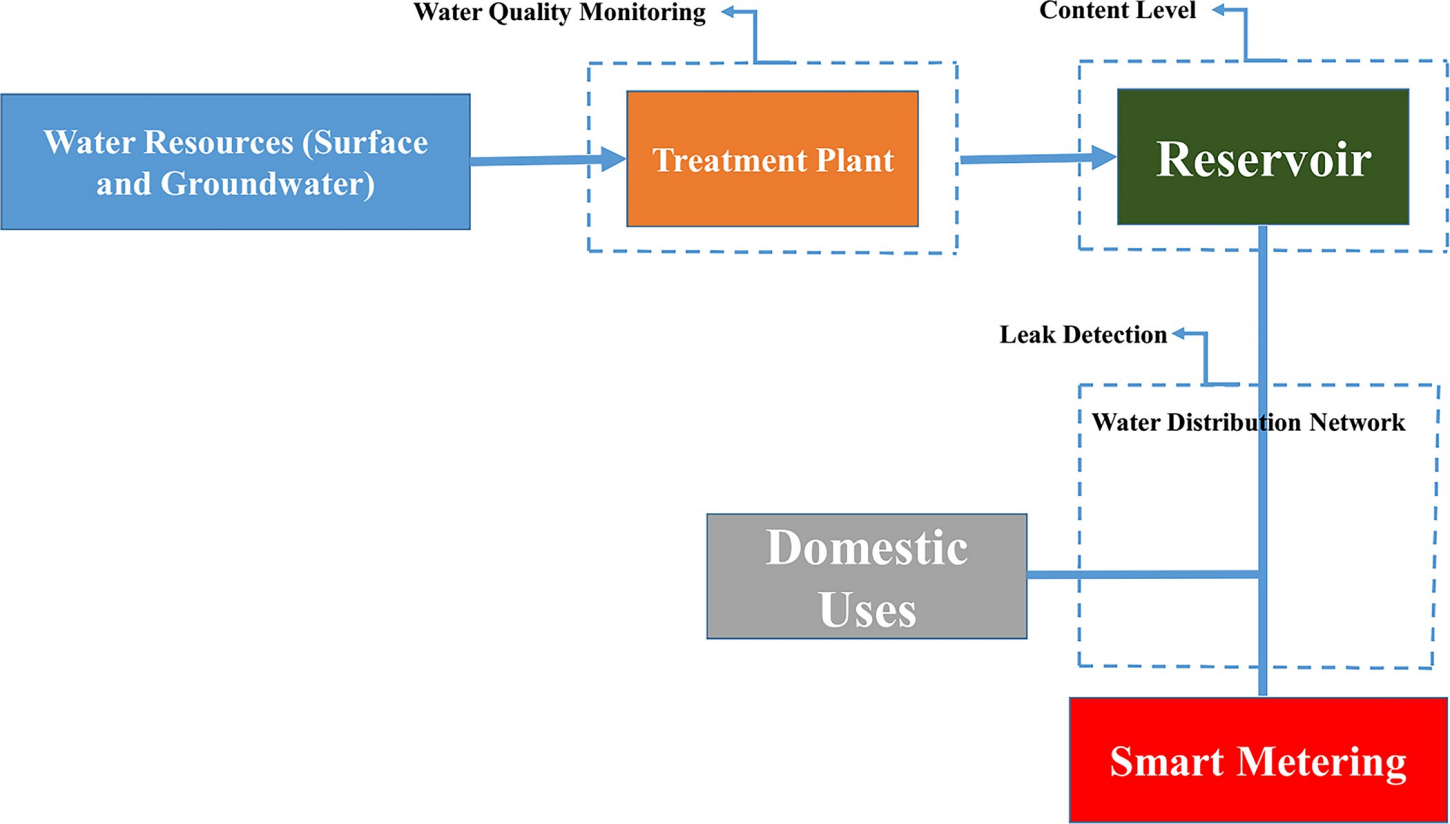
Utilization of sensing technology in water distribution systems has various applications.

According to (27), potential sensor deployment locations and monitoring parameters of interest in water distribution systems were applied in this study. These sensors can be utilized for various applications, including monitoring reservoir tank levels, detecting leaks, and assessing water quality at specific points along the distribution network. In Metje et al.’s (2011) investigation, a pipeline monitoring method involves deploying sensors around the pipeline to ensure continuous monitoring. Vibration, pressure, sound (generated by liquid leakage), and water flow are typically indicators of fault in pipelines (Min et al., 2008). The water distribution system is depicted in Fig 5. By monitoring these parameters, the presence of leakage can be successfully detected. In Stoianov et al.’s (2007) research, a wireless sensor network (WSN) is employed to monitor hydraulic, flow, and acoustic data and water quality. Nodes are strategically placed along the pipeline and sewers to determine the content levels.

**Fig 6:**
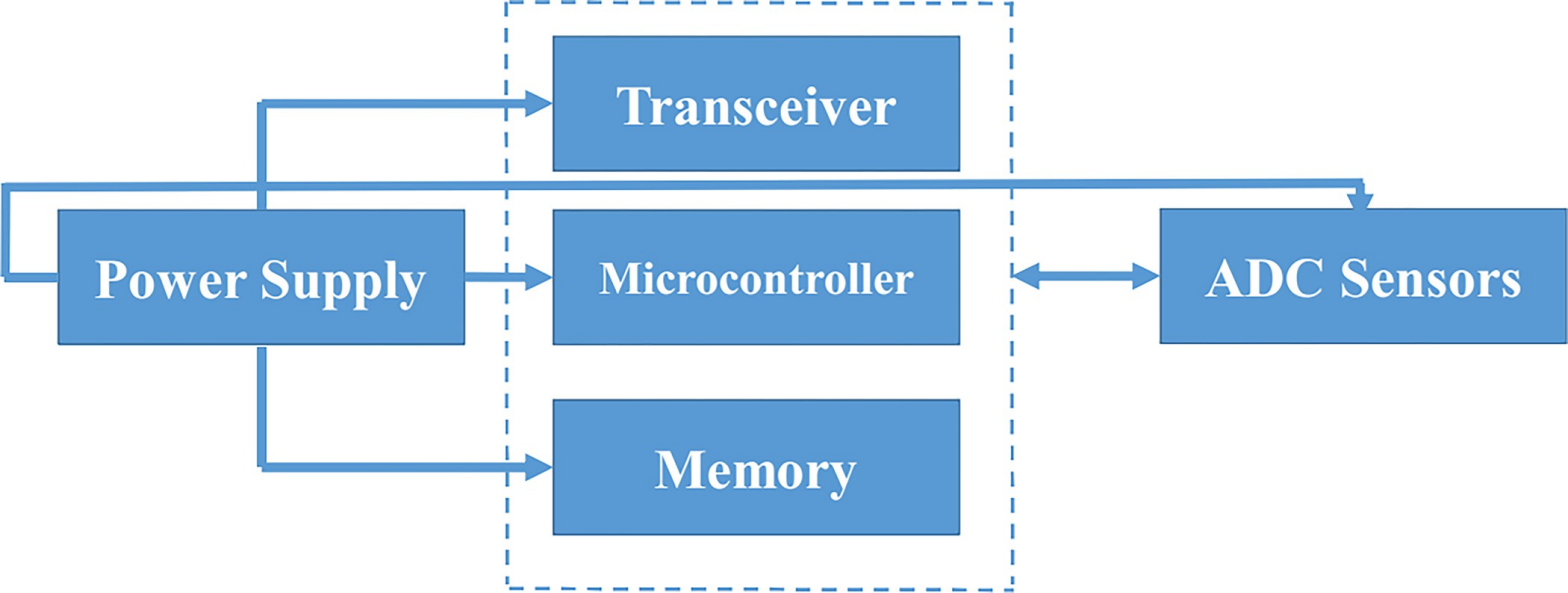
Conceptual diagram of a wireless sensor network.

Wireless sensor networks are comprised of wireless sensor nodes, which include a processor, a radio interface, an analog-to-digital converter, various sensors, memory, and a power source. The overall structure of a wireless sensor node is depicted in Fig 6.

*Singapore Case Study on IoT-Based Water Quality Assurance*. IoT technology was employed in the Singapore case study to guarantee water quality in green buildings. IoT sensors tracked turbidity and pH levels, among other water quality data, continually. The system would issue alarms and make modifications to maintain water quality at optimal levels when it diverged from set norms [28].

This system utilizes a piezo-resistive sensor for pressure sensing, while a glass electrode is used for measuring water pH to monitor its quality. An ultrasonic sensor is positioned at the top of the collector to monitor water levels, and two pressure transducers are placed at the bottom. Vibration data is collected using dual-axis accelerometers.

The gathered data is then subjected to analysis to detect leaks. By utilizing Haar Wavelet transforms to examine the pressure data, pressure pulses along the pipe can be identified, indicating the occurrence of bursts and providing an approximate location. Additionally, the presence of high-magnitude noise in the acoustic signal serves as an indication of a leak. Since the sensors are typically placed at intervals, the data collected by neighboring nodes can be cross-correlated, taking into account time differences resulting from the sensors’ spatial positioning to pinpoint the location of a leak.

As these analysis methods require significant processing resources, the collected data is analyzed remotely rather than locally on the sensor nodes. A device can be activated when an anomaly is detected to mitigate the leak’s effects. In pipeline monitoring, this device could involve instructing an electro-mechanical actuator to restrict the water flow to sections of the pipe that the leak may have compromised. Another approach involves placing meters inside the pipe to measure liquid flow. Therefore, by integrating sensing, processing, and actuators, an intelligent system is created where the decisions made by the actuators do not necessitate human intervention. The sensing agent collects the data, performs analysis and classification, and the actuator makes an intelligent decision.

#### 5.3.3 Post-Implementation analysis

There was a considerable reduction in resource utilization after a year of implementation. The energy usage was reduced to 40,000 kWh, a 20% decrease. Water consumption has also lowered by 15% to 85,000 liters. Waste generation has been reduced by 10% to 450 Kg. Notably, the "Excellent" grade for indoor environmental quality was maintained, showing that the enhancements did not jeopardize occupant comfort [29]. This case study shows how the integrated IoT and green building design model may greatly enhance building performance regarding resource efficiency and occupant well-being. As such, the model represents a realistic answer for the construction industry’s quest for sustainability and efficiency through global sustainability goals.

**Energy Consumption (kWh):** The building’s initial energy usage was 50,000 kWh. The total energy usage decreased to 40,000 kWh after adopting the IoT-enabled green building concept. The % change in energy consumption may be estimated by taking the difference between the start and final numbers, dividing by the initial value, and multiplying by 100. Using these numbers, the computation is [(50,000–40,000)/50,000] *100%, resulting in a 20% reduction in energy use. An overview of accumulated datasets is presented in Table 4.

**Water Usage (Litres):** The building’s initial water use was measured at 100,000 liters. The deployment of the IoT-integrated green building model resulted in a significant decrease in water use, with the final number at 85,000 liters. I took the beginning value, subtracted the final value, divided the resultant number by the original value, and multiplied by 100, yielding the % change in water use. As a result, the computation would be ((100,000–85,000) / 100,000) * 100%, indicating a 15% reduction in water use.

**Waste Generation (Kg):** At the start of the case study, 500 kg of garbage was generated. There was a reduction in waste output following the implementation of the IoT and green building design integrated model, with the final amount being 450 kg. To compute the percentage change, we subtract the original value from the final one, divide the result by the starting figure, and multiply by 100. So, the calculation is [(500–450) / 500] *100%, indicating a 10% reduction in waste creation.

**Table 4:** Descriptions of data collection.

Parameter	Building A Before Implementation	Building A After Implementation
**Energy Consumption (kWh)**	**50,000**	**40,000**
**Water-Usage (Liters)**	**100,000**	**85,000**
**Waste Generation (Kg)**	**500**	**450**
**IEQ Rate**	**Excellent**	**Excellent**

## 6. Results and discussion

### 6.1 Interpretation of results

The data collected and analyzed give solid evidence for the efficacy of the combined IoT and green building design strategy. Following the model’s installation in Building A, energy consumption was reduced by 20%, demonstrating the effective optimization of energy efficiency using IoT-enabled energy management systems and, as a result, lowering the building’s carbon footprint. Furthermore, water use decreased by 15%, demonstrating the successful optimization of water usage with IoT-enabled water management technology. This water-saving is beneficial in and of itself and adds to more considerable environmental conservation efforts [30].

Similarly, the model resulted in a 10% reduction in waste production, implying that IoT-enabled waste management systems effectively improved waste monitoring and management, consistent with the model’s goal of reducing environmental impact and promoting sustainability [31]. Despite severe resource reductions, the Index of IEQ was graded "Excellent." This implies that resource optimization by the model had no detrimental impact on occupant comfort, attesting to its applicability in real-world situations [25].

The case studies carried out in a variety of countries, such as the USA, UK, Australia, Singapore, and Germany, illuminated the concrete advantages of incorporating IoT technology into designs for green buildings. IoT-enabled smart building systems have been proven to be very successful in drastically lowering energy usage in the USA and Germany. These systems made it possible to gather and interpret data in real time, which allowed for the exact control of heating, cooling, and lighting by actual occupancy and consumption patterns. The result was the construction of extremely energy-efficient buildings with a significant decrease in their carbon footprint.

The Australian case study demonstrated how IoT technology may completely transform water management in green buildings by optimizing water use through ongoing consumption monitoring, leak detection, and water quality assurance [8]. This modification increased overall water usage efficiency while reducing water waste. Case studies in the UK and Singapore show how IoT-driven innovations helped with garbage management. Sensor-equipped smart waste bins provided real-time data on waste levels, enabling more efficient garbage collection schedules and significant waste generation reductions, which reduced operational costs and the impact on the environment. Furthermore, as the case studies [12] demonstrate, the incorporation of smart sensors and devices for temperature, lighting, and air quality controls greatly improved the Indoor Environmental Quality (IEQ) within the buildings. Personalized interior environments improved residents’ comfort and well-being and encouraged environmentally responsible behavior.

Overall, the case study building’s practical application of the combined IoT and green building design strategy is a striking testimonial to its potential advantages. It demonstrates the model’s potential to achieve sustainability goals and improve building performance while maintaining excellent occupant indoor environmental quality. Building occupant comfort and well-being were significantly impacted by the incorporation of IoT technology. Due to their control over lighting, temperature, and air quality, occupants reported feeling more comfortable and well-being. Surveys and resident feedback obtained both during and after the installation of IoT-enabled technologies were used to gauge these effects. Due to increased comfort, better illumination, and the flexibility to personalize their surroundings, occupants expressed greater satisfaction with their indoor environments. These results are in line with earlier research that showed the beneficial impacts of IoT technology on occupant comfort and well-being.

### 6.2 Implications for green building and IoT industry

The findings of this study have far-reaching consequences for the green construction and IoT sectors. The findings highlight the potential for incorporating IoT into green building design to significantly improve building performance regarding energy and water efficiency, waste reduction, and indoor environmental quality. One of the most important aspects of environmental preservation is the incorporation of IoT technology. Through the analysis of real-time occupancy and environmental data, IoT-enabled smart building systems improve energy efficiency, leading to fewer carbon emissions and energy consumption. Another advantage is that IoT-based devices can conserve water by monitoring and optimizing water use and identifying leaks. This lessens the impact of water waste on the environment.

Real-time monitoring made possible by IoT sensors also revolutionizes waste management by enabling effective waste collection schedules and lower operating expenses. Additionally, by controlling lighting, humidity, temperature, and air quality, IoT improves interior environmental quality and eventually increases occupant comfort and well-being. Finally, by using IoT sensors for predictive maintenance, building systems can last longer, require fewer resource-intensive replacements, and produce less waste. The model’s proven real-world performance offers the green construction sector a viable and effective way of reaching sustainability goals. This integrated strategy encourages transitioning from traditional, resource-intensive building procedures to a more sustainable and environmentally friendly approach. In terms of the IoT sector, the study emphasizes the importance of IoT in the green construction industry and its potential contribution to sustainable urban development.

According to the study, green building design represents a promising market for IoT developers and service providers since their solutions may address actual, real-world difficulties. Unexpected results could include the necessity to successfully balance environmental trade-offs, positive occupant behavior changes, and synergistic benefits The research also emphasizes the need for IoT solutions, especially customized to green building requirements, such as energy-efficient devices and practical data processing tools. Furthermore, incorporating IoT into green building design has far-reaching consequences for legislators, urban planners, and environmental activists. The method supports a transition to smart, sustainable cities by demonstrating the potential of advanced technology in tackling significant environmental concerns and encouraging sustainable living [22].

## 7. Conclusion

This study draws numerous vital findings concerning the feasibility of implementing IoT technology into green building design. Resource optimization is one of the most successful outcomes. The case study revealed that the IoT-enabled green building concept significantly boosted resource efficiency. This was proved by a 20% drop in energy usage, a 15% decrease in water consumption, and a 10% decrease in trash generation. This demonstrates IoT technology’s importance in reaching resource efficiency goals in green buildings. The quality of the building’s internal atmosphere remained maintained even with reduced resource consumption. This shows that using IoT technology to balance resource efficiency and occupant comfort in green buildings is possible. Aside from maintaining a high-quality indoor atmosphere, the model’s practical application in a real-world setting indicates its scalability.

This implies that the approach may be applied in more buildings or on a city-wide scale, adding to the sustainability of urban growth. The results have consequences for the industry as well. They emphasize a prospective market for IoT technology in the green building sector and the potential for green building practices to boost construction sustainability. Thus, incorporating IoT technology into green building design has enormous potential for increasing building efficiency, achieving environmental sustainability goals, and stimulating the creation of intelligent, sustainable cities.

The research has practical implications in two main areas. Additionally, it thoroughly examines the obstacles faced in implementing green building (G.B.) projects in Turkey, providing a comprehensive understanding of these barriers. Moreover, it clarifies the perspectives of public agency representatives and professionals working in private entities regarding the significance of these barriers. This more profound understanding of the barriers can help policymakers and construction practitioners develop well-informed strategies to promote green practices in China and other developing countries with similar socio-economic conditions. Furthermore, the in-depth analysis of these barriers can benefit foreign investors interested in investing in G.B. projects in China. By better understanding the G.B. industry in China, they can make more realistic investment decisions.

However, it is essential to note that the study has limitations. There were obstacles and difficulties in integrating IoT technology into the design of green buildings. A prominent obstacle was the upfront expenses associated with setting up IoT infrastructure and installing devices, which were frequently viewed as a substantial financial commitment. However, the long-term savings in energy consumption, upkeep, and operational efficiency that IoT devices provided helped to offset this cost.

Concerns about data security and privacy were also very important because IoT devices required the gathering and sharing of sensitive data. Strong security procedures and encryption techniques were put in place to protect data integrity and privacy to allay these worries. The requirement for certain knowledge and abilities to successfully manage and run IoT-enabled technologies presented another difficulty. Training was necessary for building management employees to handle and comprehend the data produced by IoT devices.

In addition, there were problems with compatibility when combining IoT solutions with pre-existing building systems. Thorough preparation and compatibility evaluations were required to guarantee a smooth integration Notwithstanding these difficulties, IoT technology is a potential strategy for sustainable building design because its overall advantages, like improved occupant comfort and energy efficiency, exceeded the early drawbacks.

Although more significant than the recommended value for proper factor analysis, the sample size used in the research is still relatively small. Increasing the sample size in future studies could yield more reliable results. Additionally, future research can focus on expanding the participant demographics to ensure a more balanced distribution. While this study primarily focused on barriers to G.B. projects, future investigations could explore the barriers and the driving factors in different countries.

Furthermore, influential factors on IEQ will be analyzed by Principal Component Analysis (PCA) and Hierarchical Cluster Analysis (HCA). Ultimately, this index would be predicted by various Machine Learning (ML) models (i.e., Evolutionary Polynomial Regression [EPR], Deep Learning [DL], Random Forest [R.F.], Support Vector Machine [SVM]) through the process of G.B. design by IoT.

### 7.1 Future studies

Future research studies could improve the organization and coherence of the transition from outlining the limitations of the study to suggesting future research directions. Based on our study’s findings, numerous significant future research objectives and areas for development in green building design use IoT technology. First, sophisticated IoT applications, especially for optimizing renewable energy sources like solar and wind power, can improve energy efficiency. Understanding how IoT affects occupant behavior and well-being, especially in personalized IoT-driven settings, can inform human-centric design

To secure building systems and tenant data, IoT data collection and processing must be thoroughly investigated for cybersecurity and privacy issues. Further research is needed to standardize and interoperate IoT devices and systems for scalability and acceptance in green building design.

A detailed cost-benefit analysis will help stakeholders decide on the financial and long-term benefits of IoT integration in green buildings. Governments and regulators can promote sustainability by studying how policies and regulations affect IoT integration.

Finally, architectural, design, and building management professionals require specific education and training to use IoT’s promise in green building design. These programs can equip practitioners for the changing landscape of IoT technologies in sustainability and environmental preservation. IoT technology in green building design is relevant globally but requires regional and local considerations. Sustainability, energy efficiency, and environmental preservation are universal values, but obstacles and priorities vary. Climate, legal frameworks, resource availability, cultural factors, economic factors, and infrastructure readiness all affect IoT-enabled green building solutions. Extreme climates may optimize HVAC, while water scarcity zones may use IoT to manage water. Local building codes must be followed, and economic concerns may affect IoT implementations.

## Supporting information

S1 Dataset (XLSX)
